# Simultaneous demultiplexing and steering of multiple orbital angular momentum modes

**DOI:** 10.1038/srep15406

**Published:** 2015-10-27

**Authors:** Shuhui Li, Jian Wang

**Affiliations:** 1 Wuhan National Laboratory for Optoelectronics, School of Optical and Electronic Information, Huazhong University of Science and Technology, Wuhan 430074, Hubei, China

## Abstract

We present a simple scheme to perform simultaneous demultiplexing and steering of multiple orbital angular momentum (OAM) modes using a single complex phase mask. By designing the phase mask, the propagation directions of demultiplexed beams can be arbitrarily steered. System experiments using orthogonal frequency-division multiplexing 32-ary quadrature amplitude modulation (OFDM-32QAM) signals over two OAM modes are carried out by using a two-mode complex phase mask. Moreover, demultiplexing of sixteen OAM modes and arbitrary demultiplexed beam steering are also demonstrated in the experiment.

In the recent years, space-division multiplexing (SDM) has been proposed as a promising technology to facilitate dramatic increase in transmission capacity by exploring the spatial domain of light waves. SDM with few-mode fiber (FMF) and multi-core fiber (MCF) has been widely investigated in fiber optical transmission systems^1^,^2^,^3^. Alternatively, SDM using orbital angular momentum (OAM) also shows the potential to increase the transmission capacity and spectral efficiency by multiplexing multiple orthogonal OAM modes^4^. OAM beam is characterized by a helical phase form of exp(*ilφ*) (*l* = 0, ±1, ±2, …), where *l* is the topological charge number and *φ* refers to the azimuthal angle^5^. OAM modes with different *l* values are intrinsically orthogonal and separable with each other. Very recently, OAM multiplexing in free space has achieved 230-bit/s/Hz spectral efficiency and 1.036-Pbit/s transmission capacity^6^,^7^. Terabit-scale OAM transmission over 1.1-km vortex fiber has also been demonstrated^8^.

For an OAM multiplexing system, it is believed that the most critical components are multiplexer and demultiplexer. There have been many attempts to (de)multiplex OAM modes, including the use of cylindrical lens mode converters, fiber mode couplers, q-plates, spiral phase plates, spatial light modulators (SLM), metamaterials-based phase plate and silicon integrated devices^9^,^10^,^11^,^12^,^13^,^14^. Among all the methods, SLM is the most widely used one owing to its flexible programming control. However, in most of the previous SLM-assisted OAM (de)multiplexing experiments^4^,^6^,^7^, one SLM together with some accessary elements (e.g. beam splitter, lens, polarizer, half-wave plate) was used to demultiplex one desired OAM mode. Hence, multiple SLMs with a large number of accessary elements would be required to enable simultaneous multi-OAM demultiplexing, resulting in considerable increase of the system complexity. Moreover, one would also expect to flexibly control the beam direction after OAM demultiplexing. In this scenario, a laudable goal would be to develop a simple and robust approach to simultaneously demultiplexing all OAM modes and arbitrarily steering the direction of the demultiplexed beams^15^.

In this paper, we propose a simple method to simultaneously demultiplex multiple OAM modes accompanied by arbitrary beam steering with a single complex phase mask. The directions of demultiplexed beams can be arbitrarily controlled by designing the phase mask. A two-OAM multiplexing system is carried out. Orthogonal frequency-division multiplexing 32-ary quadrature amplitude modulation (OFDM-32QAM) signals are employed over two OAM modes which are simultaneously demultiplexed and steered by the designed complex phase mask. Moreover, demultiplexing of sixteen OAM modes and arbitrary demultiplexed beam steering are also demonstrated.

## Results

### Concept and principle

**Figure 1:**
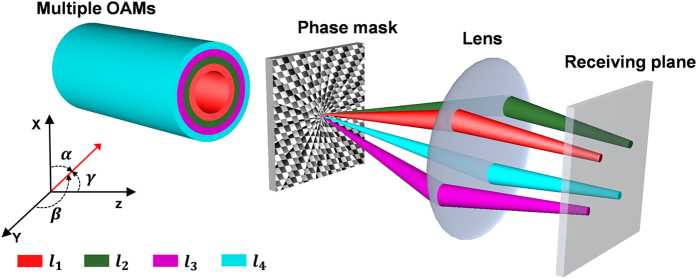
Concept and principle of simultaneous multi-OAM demultiplexing and arbitrary demultiplexed beam steering with a single phase mask and lens.

The concept and principle of simultaneous multi-OAM demultiplexing and steering are shown in Fig. 1. Input multiple co-propagating OAM modes are simultaneously demultiplexed by a specially designed complex phase mask. This phase mask converts multiple OAM beams to multiple Gaussian-like beams and delivers them along different directions. The direction (α,  β,  γ) of each demultiplexed beam can be precisely steered by designing the phase mask. After passing through a lens, the demultiplexed beams are focused on receiving plane with a desired distribution. As an example shown in Fig. 1, the complex phase mask is designed to achieve a parallelogram-shape spatial distribution of four demultiplexed beams. At the receiving plane, demultiplexed beams can be collected by single-mode fiber (SMF) array for detection. Compared with conventional scheme employing multiple SLMs loaded with multiple helical phase masks and lots of beam splitters (BS) and lenses to achieve simultaneous multi-OAM demultiplexing, only single phase mask and single lens are employed in the proposed approach. Several distinct features in Fig. 1 are as follows: 1) simultaneous multi-OAM demultiplexing with a single complex phase mask; 2) arbitrary beam steering after OAM demultiplexing; 3) scalable to demultiplexing of a large number of OAM modes without adding extra elements. In addition, the inverse process in Fig. 1 corresponds to the multi-OAM generation and multiplexing.

**Figure 2:**
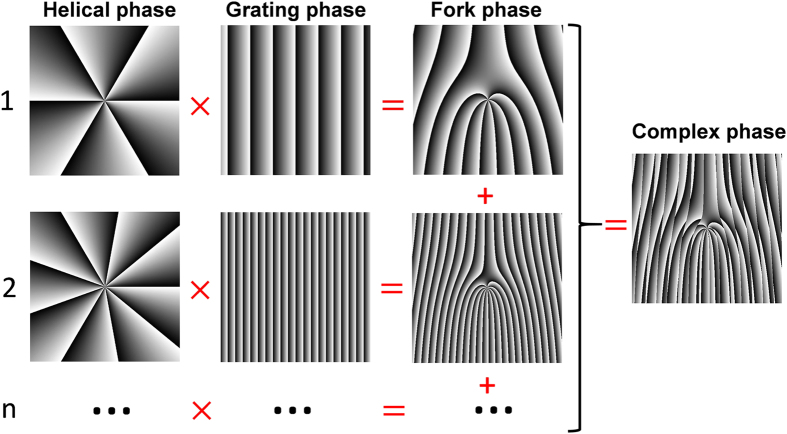
Method of generating the complex phase mask.

Figure 2 illustrates the method of generating the complex phase mask for multi-OAM (de)multiplexing accompanied by beam steering. As well known, an OAM (*l*_*i*_) beam can be converted from or back-converted to a Gaussian-like beam by a helical phase mask termed as exp(*il*_*i*_*φ*) or exp(−*il*_*i*_*φ*). The helical phase mask only changes the transverse phase structure without varying the propagation direction of the incident beam. In order to convert (back-convert) an OAM beam from (to) a Gaussian-like beam in a specific propagation direction, one can multiply the helical phase mask by a blazed grating phase mask termed as 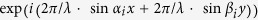 and get a resultant conventional fork phase mask expressed as


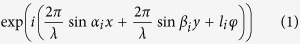


where *x* is the horizontal coordinate, y is the vertical coordinate, *α*_*i*_ is the diffraction angle of horizontal direction, and *β*_*i*_ is the diffraction angle of vertical direction. By controlling the diffraction angle or the period of the blazed phase grating, one can easily steer the propagation direction of the converted or back-converted beam. The specific complex phase mask for multi-OAM (de)multiplexing and steering can be constructed by adding multiple conventional fork phase masks with different *α*_*i*_, *β*_*i*_ and *l*_*i*_ together as follows


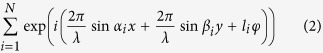


where *N* is the number of OAM beams. The propagation direction corresponding to each OAM mode (*l*_*i*_) can be flexibly controlled by choosing *α*_*i*_ and *β*_*i*_.

### Experimental setup

**Figure 3:**
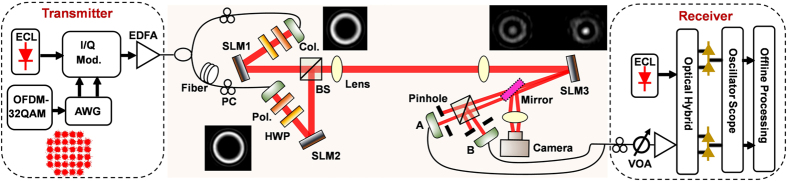
Experiment setup for simultaneous demultiplexing of two OAM modes and demultiplexed beam steering with a single complex phase mask. ECL: external cavity laser; AWG: arbitrary waveform generator; I/Q Mod.: in-phase/quadrature modulator; EDFA: erbium-doped fiber amplifier; PC: polarization controller; Col.: collimator; Pol.: polarizer; HWP: half-wave plate; SLM: spatial light modulator; VOA: variable optical attenuator; BS: beam splitter.

The experiment setup is displayed in Fig. 3. At the transmitter, an external cavity laser (ECL) at 1550 nm is fed into an optical I/Q modulator to carry OFDM QAM signal. An arbitrary waveform generator (AWG) is employed to produce electrical OFDM 32-QAM signal. The signal is then split into two paths relatively delayed with fiber, and then projected onto two SLMs loaded with helical phase masks to generate two OAM modes. After a free-space transmission link, another SLM loaded with a specially designed complex phase mask is used for simultaneous multi-OAM demultiplexing and beam steering. The demultiplexed beams are captured by a camera after passing through a lens or split into two paths for easy measurement and coupled into two single-mode fibers for coherent detection. A variable optical attenuator (VOA) is employed to adjust the optical signal-to-noise ratio (OSNR) before the receiver. At the receiver, a local oscillator is mixed with the received signals in a coherent receiver. The received radio frequency (RF) signals are fed into a real-time scope and processed off-line with MATLAB program.

### Experiment results

**Figure 4:**
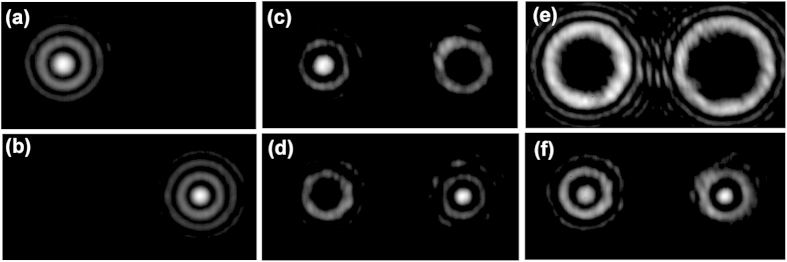
Measured intensity profiles. (**a**,**b**) OAM_−6_ and OAM_−9_ demultiplexing with conventional fork phase mask and single incident OAM mode. (**c**,**d**) OAM_−6_ and OAM_−9_ demultiplexing with specially designed two-mode complex phase mask and single incident OAM mode. (**e**) A Gaussian beam incident onto the two-mode complex phase mask. (**f**) Two OAM modes incident onto the two-mode complex phase mask (simultaneous demultiplexing of two OAM modes accompanied by beam steering).

We first design a two-mode complex phase mask for OAM_−6_ and OAM_−9_ simultaneous demultiplexing and beam steering. SLM1 and SLM2 loaded with fork phase masks are used to generate OAM_−6_ and OAM_−9_, respectively. The doughnut-shape intensity profiles of OAM_−6_ and OAM_−9_ are depicted in the insets of Fig. 3. Then OAM_−6_ and OAM_−9_ are combined together by a beam splitter. When using conventional fork phase masks (i.e. combined helical phase and grating phase masks shown in Fig. 2) for demultiplexing, the demultiplexed intensity profiles of OAM_−6_ and OAM_−9_ with single incident OAM mode (OAM_−6_ or OAM_−9_) are shown in Fig. 4(a,b). One can clearly see high-intensity bright spots at the center of beams. The two demultiplexed beams are at different locations due to different grating periods adopted in the demultiplexing fork phase masks. When using specially designed complex phase mask according to Fig. 2 for demultiplexing, the demultiplexed intensity profiles of OAM_−6_ and OAM_−9_ with single incident OAM mode (OAM_−6_ or OAM_−9_) are shown in Fig. 4(c,d), from which one can confirm successful demultiplexing of OAM_−6_ and OAM_−9_. The locations of demultiplexed beams are the same as the case using conventional fork phase masks for individual demultiplexing. Moreover, one can also see additional ring-shape intensity profiles in Fig. 4(c,d). These ring-shape intensity profiles are not expected, but inevitable in this simple scheme, which can be briefly explained as follows. As illustrated in Fig. 2, the specially designed complex phase mask capable of demultiplexing two incident OAM modes (OAM_−6_ and OAM_−9_) is actually the combination of two conventional fork phase masks with the first one for OAM_−6_ demultiplexing (Fig. 4(a)) and the second one for OAM_−9_ demultiplexing (Fig. 4(b)). For single incident OAM_−6_ (OAM_−9_) mode, the first (second) conventional fork phase mask component in the complex phase mask enables the OAM_−6_ (OAM_−9_) demultiplexing and its steering on the left (right), while the second (first) conventional fork phase mask component in the complex phase mask also functions on the OAM_−6_ (OAM_−9_) mode but updates its topological charge number to another non-zero value, resulting in the unwanted additional ring-shape intensity profile on the right (left), as shown in Fig. 4(c,d). These additional intensity profiles can cause energy loss. However, they have little impact on demultiplexing due to ring-shape intensity distributions which are not coupled into single mode fiber. To assess the insertion loss of the two-mode complex phase mask, we measure the received demultiplexing power at point A and B. For OAM_−6_, the received power is −7.3 dBm and −10.69 dBm for fork phase mask and two-mode complex phase mask demultiplexing, respectively. For OAM_−9_, the received power is −9.54 dBm and −12.95 dBm respectively. The insertion loss is less than 3.41 dB, featuring similar loss level to perform multiple OAM demultiplexing with multiple SLMs loaded with multiple helical phase masks and lots of beam splitters (BS). In contrast, less components are required here. The method proposed in^14^ can simultaneously separate multiple OAM modes with a small loss. But the position of each demultiplexed beam is not flexibly controlled. For a Gaussian beam incident onto the complex phase mask, two separated OAM modes are generated as shown in Fig. 4(e). For two OAM modes incident onto the complex phase mask, simultaneous demultiplexing of two OAM modes accompanied by beam steering is achieved as shown in Fig. 4(f). The two demultiplexed beams propagating along different directions as shown in Fig. 3, can be coupled into single mode fiber array for detection. We use two single mode fiber and a beam splitter to emulate the function of a fiber array.

**Figure 5:**
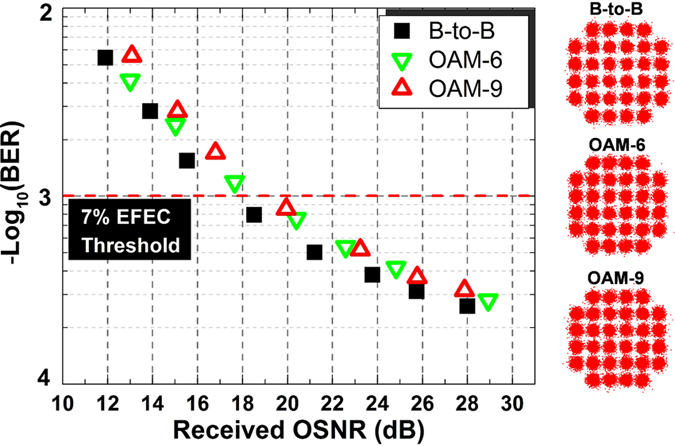
Measured BER curves and constellations for OFDM-32QAM carrying OAM_−6_ and OAM_−9_ (d,e) multiplexing.

We then perform the system experiments using 40.31-Gbit/s OFDM-32QAM signals over two OAM modes. We measure the BER performance of OAM_−6_ and OAM_−9_ modes carrying 40.31-Gbit/s OFDM-32QAM signals demultiplexed by the specially designed two-mode complex phase mask. As shown in Fig. 5, the observed optical signal-to-noise ratio (OSNR) penalties are less than 1.8 dB at a bit-error rate (BER) of 1e–3 (forward error correction (FEC) threshold). The insets of Fig. 5 also plot constellations of 32QAM.

**Figure 6:**
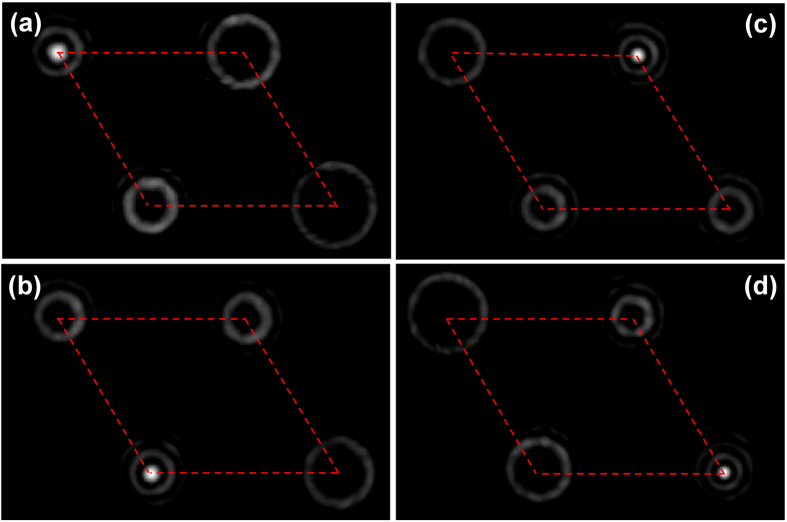
Measured intensity profiles for (a) OAM_−6_, (b) OAM_−9_, (c) OAM_−12_ and (d) OAM_−15_ demultiplexing. The four demultiplexed beams are steered in a parallelogram shape.

We also design a four-mode complex phase mask to demultiplex OAM_−6_, OAM_−9_, OAM_−12_ and OAM_−15_. To show the ability of arbitrary demultiplexed beam steering, the complex phase pattern is designed to achieve a parallelogram-shape spatial distribution of four demultiplexed beams. The measured demultiplexed beams intensity profiles are shown in Fig. 6. The four demultiplexed beams are steered in a parallelogram shape consistent with the design parameters. Flexible demultiplexed beam steering is very useful especially for fiber array receiving multiple demultiplexed beams. Generally, each location of input port of a commercial fiber array is fixed. The fixed port is not convenient for multiple incident beams coupling since the location of each port might be misaligned with the incident beam. However, if the direction of each incident beam can be flexibly controlled, the coupling between multiple beams and fiber array could become easier.

**Figure 7:**
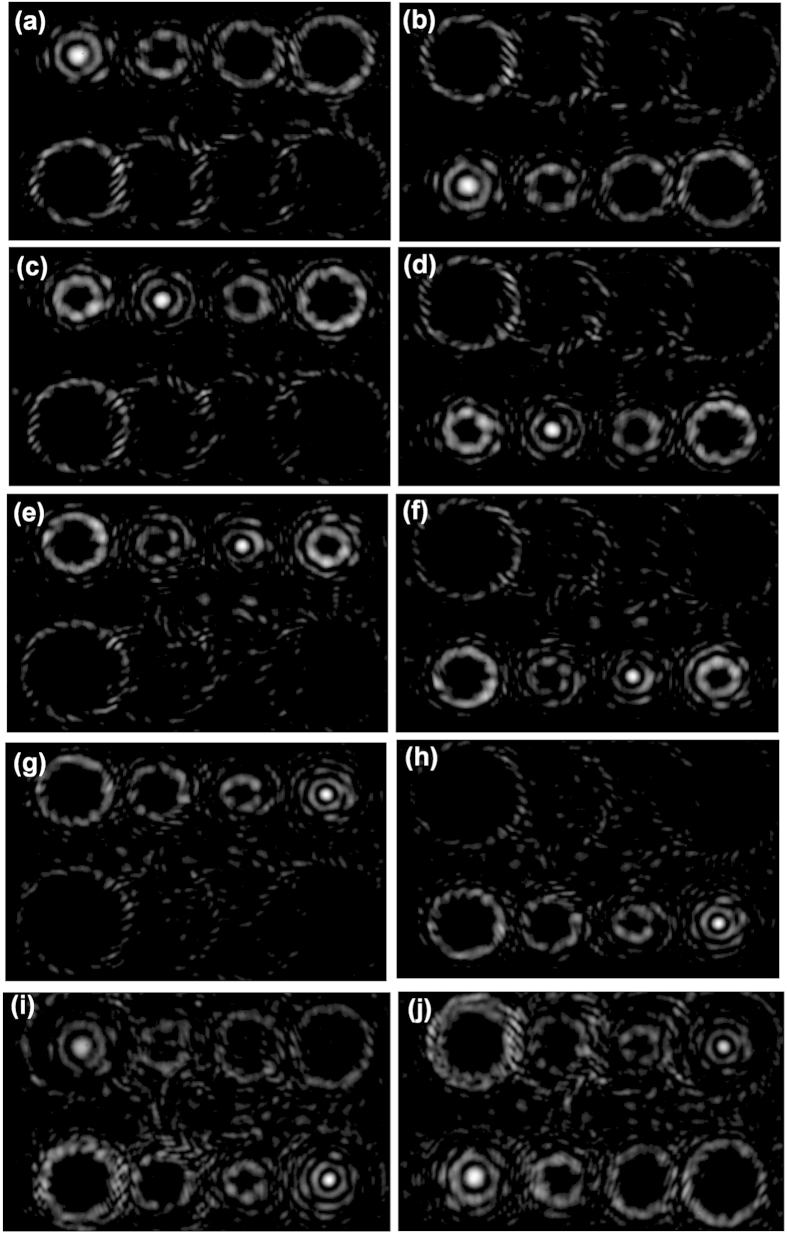
Measured intensity profiles for (a) OAM_−6_, (b) OAM_6_, (c) OAM_−9_, (d) OAM_9_, (e) OAM_−12_, (f) OAM_12_, (g) OAM_−15_, (h) OAM_15_, (i) OAM_−6_, OAM_15_ and (j) OAM_−15_, OAM_6_ demultiplexing. The eight demultiplexed beams are steered in two rows (rectangular shape).

We further demonstrate eight OAM demultiplexing and steering (OAM_−6_, OAM_−9_, OAM_−12_, OAM_−15_, OAM_6_, OAM_9_, OAM_12_, OAM_15_) with an eight-mode complex phase mask. The eight demultiplexed beams are steered in two rows (rectangular shape) by the complex phase mask. For single incident OAM mode, demultiplexed intensity profiles are measured as shown in Fig. 7(a–h), while for two incident OAM modes, the measured intensity profiles are shown in Fig. 7(i,j). One can clearly see that the designed complex phase mask successfully enables eight OAM demultiplexing and the eight demultiplexed beams are distributed in two rows (rectangular shape) as expected.

**Figure 8:**
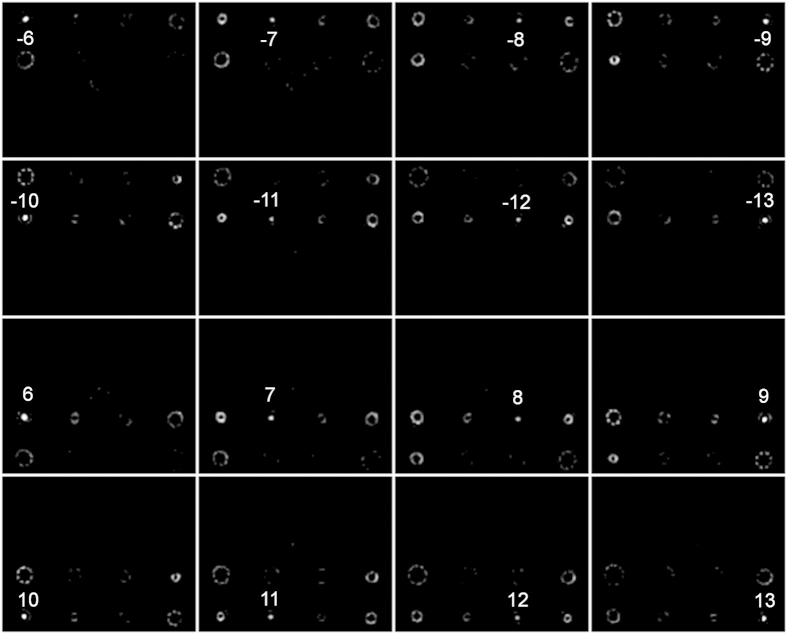
Measured intensity profiles for OAM *l* = ±6, ±7, ±8, ±9, ±10, ±11 demultiplexing. The sixteen demultiplexed beams are steered in four rows (rectangular shape).

In the above demonstrations, the minimum interval of different OAM beams is 3. Actually, the interval can be as small as 1. Moreover, the number of OAM modes for simultaneous demultiplexing and steering can be further increased and the distributions of demultiplexed beams can be more complicated. We design a specific complex phase mask for demultiplexing and steering of sixteen OAM beams with *l* = ±6, ±7, ±8, ±9, ±10, ±11, ±12, ±14. The sixteen demultiplexed beams are steered in four rows (rectangular shape) by the complex phase mask. As shown in Fig. 8, one can clearly see that the designed complex phase mask successfully enables sixteen OAM demultiplexing and the sixteen demultiplexed beams are distributed in four rows (rectangular shape) as expected.

**Figure 9:**
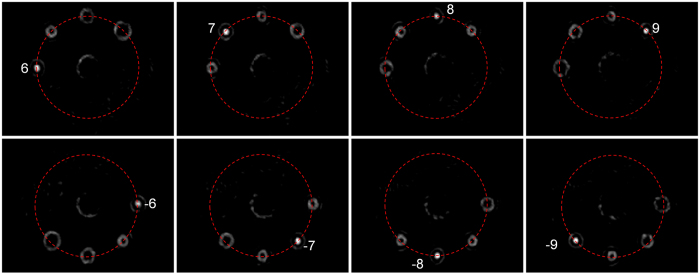
Measured intensity profiles for OAM *l* = ±6, ±7, ±8, ±9 demultiplexing. The eight demultiplexed beams are steered in a circular shape.

Additionally, we also design a specific complex phase mask to realize demultiplexing of OAM *l* = ±6, ±7, ±8, ±9 with circular-shape beam steering of demultiplexed beams. The measured demultiplexed intensity profiles are shown in Fig. 9. One can clearly see that the eight demultiplexed beams are steered in a circular shape as expected.

## Discussion

We experimentally demonstrate simultaneous demultiplexing of multiple OAM modes accompanied by arbitrary beam steering by exploiting a single complex phase mask. Simultaneous multiple OAM demultiplexing can reduce the complexity and the number of accessary elements of the system. Moreover, arbitrary beam steering could be also useful in wide applications, such as the coupling between fiber array and multiple beams. The demonstrated scheme of simultaneous demultiplexing and steering of multiple OAM modes might facilitate flexible and robust OAM (de)multiplexing systems for OAM communications.

## Methods

We employ a phase-only spatial light modulator (SLM) with a resolution of 1920 × 1080 pixel and 8 μm pixel pitch to demonstrate the simultaneous demultiplexing and steering of multiple OAM modes in the experiment. The SLM is optimized for use at a wavelength of 1550 nm. The designed phase masks loaded onto SLM are Bitmap figures generated by a MATLAB program.

## Additional Information

**How to cite this article**: Li, S. and Wang, J. Simultaneous demultiplexing and steering of multiple orbital angular momentum modes. *Sci. Rep.* **5**, 15406; doi: 10.1038/srep15406 (2015).
